# Effects of intra-aortic balloon pump on cerebral blood flow during peripheral venoarterial extracorporeal membrane oxygenation support

**DOI:** 10.1186/1479-5876-12-106

**Published:** 2014-04-27

**Authors:** Feng Yang, Zai-shen Jia, Jia-lin Xing, Zheng Wang, Yuan Liu, Xing Hao, Chun-jing Jiang, Hong Wang, Ming Jia, Xiao-tong Hou

**Affiliations:** 1 Department of Extracorporeal Circulation, Center for Cardiac Intensive Care, Beijing Anzhen Hospital, Capital Medical University, 2 Anzhen Rd, Chaoyang District, Beijing 100029, P.R. China; 2 Center for Cardiac Intensive Care, Beijing Anzhen Hospital, Capital Medical University, Beijing, China

**Keywords:** Intra-aortic balloon pump, Venoarterial extracorporeal membrane oxygenation, Cerebral blood flow, Transcranial doppler ultrasound

## Abstract

**Background:**

The addition of an intra-aortic balloon pump (IABP) during peripheral venoarterial extracorporeal membrane oxygenation (VA ECMO) support has been shown to improve coronary bypass graft flows and cardiac function in refractory cardiogenic shock after cardiac surgery. The purpose of this study was to evaluate the impact of additional IABP support on the cerebral blood flow (CBF) in patients with peripheral VA ECMO following cardiac procedures.

**Methods:**

Twelve patients (mean age 60.40 ± 9.80 years) received VA ECMO combined with IABP support for postcardiotomy cardiogenic shock after coronary artery bypass grafting. The mean CBF in the bilateral middle cerebral arteries was measured with and without IABP counterpulsation by transcranial Doppler. The patients provided their control values. The mean CBF data were divided into two groups (pulsatile pressure greater than 10 mmHg, P group; pulsatile pressure less than 10 mmHg, N group) based on whether the patients experienced cardiac stun. The mean cerebral blood flow in VA ECMO (IABP turned off) alone and VA ECMO with IABP support were compared using the paired *t* test.

**Results:**

All of the patients were successfully weaned from VA ECMO, and eight patients survived to discharge. The addition of IABP to VA ECMO did not change the mean CBF (251.47 ± 79.28 ml/min vs. 251.30 ± 79.47 ml/min, *P* = 0.96). The mean CBF was higher in VA ECMO alone than in VA ECMO combined with IABP support in the N group (257.68 ± 97.21 ml/min vs. 239.47 ± 95.60, *P* = 0.00). The addition of IABP to VA ECMO support increased the mean CBF values significantly compared with VA ECMO alone (261.68 ± 82.45 ml/min vs. 244.43 ± 45.85 ml/min, *P* = 0.00) in the P group.

**Conclusion:**

These results demonstrate that an IABP significantly changes the CBF during peripheral VA ECMO, depending on the antegrade blood flow by spontaneous cardiac function. The addition of an IABP to VA ECMO support decreased the CBF during cardiac stun, and it increased CBF without cardiac stun.

## Background

Venoarterial extracorporeal membrane oxygenation (VA ECMO) is increasingly used in various clinical acute settings such as extracorporeal cardiopulmonary resuscitation (ECPR) or cardiogenic shock refractory to conventional treatment. There is concern that VA ECMO might change the CBF and impair cerebral autoregulation, resulting in neurologic dysfunction [1-8]. A clinical study demonstrated that intra-aortic balloon pump (IABP) support in patients after cardiac surgery caused a considerable increase in the flow velocity in the middle cerebral artery [9]. In clinical practice, the addition of an IABP during VA ECMO support is believed to improve coronary bypass graft flows and cardiac function in critically ill patients [10,11]. IABP has been successfully applied as an adjunct to VA ECMO in patients with cardiogenic shock [12]. Despite the beneficial effects on cardiac performance, the effects of this combination on the CBF are conflicting. One recent clinical experience paper suggested that an IABP obstructed VA ECMO flow in the aorta in patients on peripheral VA ECMO [13]. End-diastolic reversal of blood flow in the cerebral arteries has been observed in some patients with IABP [9], which likely suggests that the flow velocity in the right middle cerebral artery did not increase with IABP. Transcranial Doppler ultrasound (TCD) is a noninvasive technique that allows for repeatable bedside assessments of the CBF velocity (CBFV) during cardiac and vascular surgery [14]. Previous studies showed a positive relationship between the CBF and CBFV [15]. The effect of an IABP on the CBF during VA ECMO support has not been examined by TCD in humans.

We hypothesized that an IABP might change the CBF during peripheral VA ECMO in adult patients. In the current study, we measured the blood flow of the bilateral middle cerebral arteries with TCD and found it might be associated with the cardiac functional state.

## Materials and methods

This study was approved by the institutional ethics committee of Beijing Anzhen Hospital, Capital Medical University. Informed consent was obtained for each patient. The clinical data were prospectively collected for all of the patients undergoing VA ECMO combined with IABP support at the Beijing Anzhen Hospital, and the data were stored in an electronic database.

### Patients

**Figure 1:**
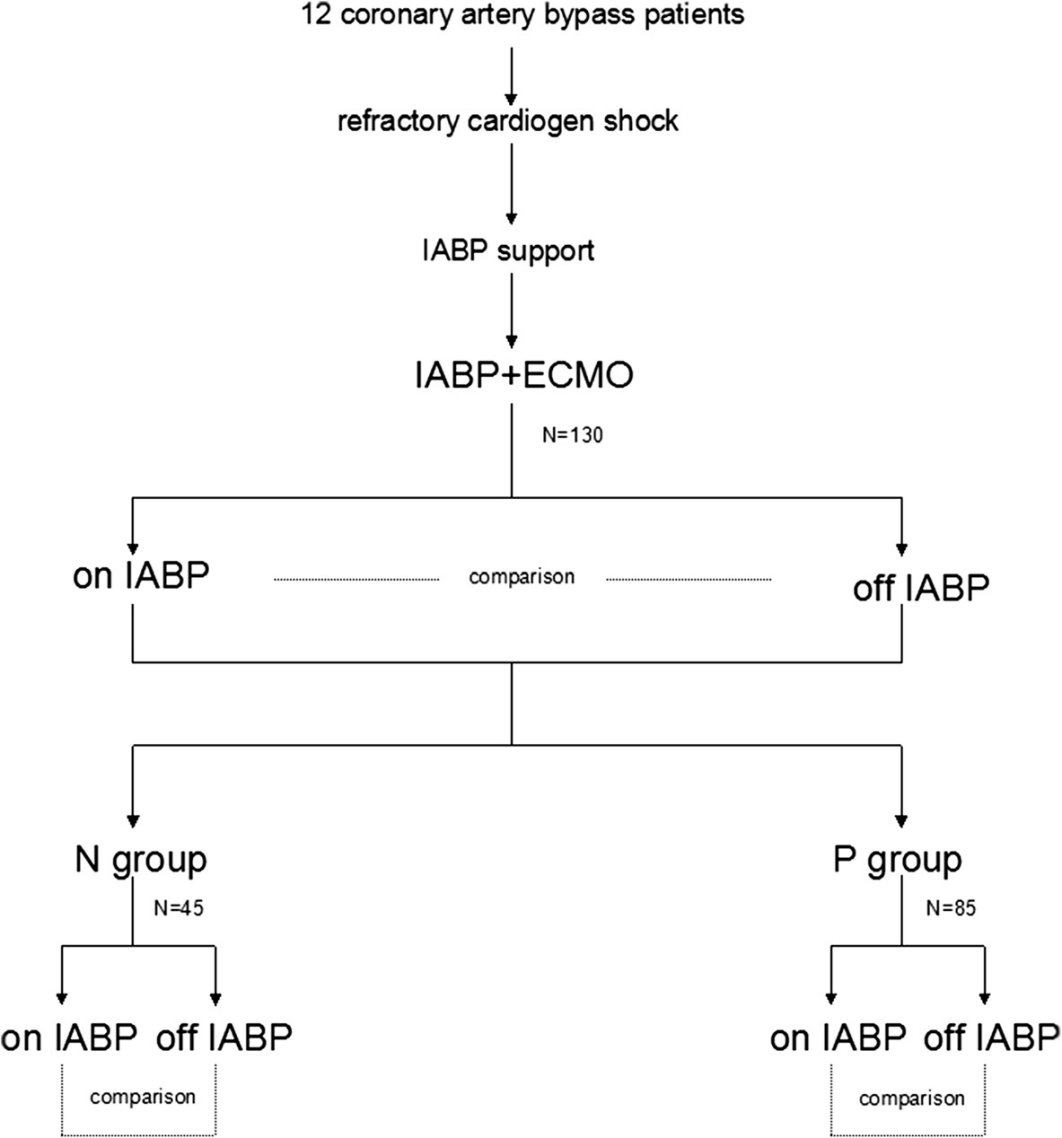
**Scheme showing the study protocol.** N group, pulsatile pressure ≤ 10 mmHg; P group, pulsatile pressure greater than 10 mmHg.

Between January 2011 and August 2012, 12 consecutive adult patients (mean age 60.40 ± 9.80 years, ranging from 44 to 76 years; 3 females, 25%) receiving VA ECMO and IABP support for refractory postcardiotomy cardiogenic shock after coronary artery bypass graft were enrolled in the study. No patients had a neurologic disease or any cerebral vascular disorders, as confirmed by pre-operative brain computed tomography, ultrasonography, and magnetic resonance imaging. The indications for VA ECMO support included clinical criteria of postoperative refractory cardiogenic shock, signs of end-organ failure, anaerobic metabolism, severe metabolic acidosis, and oliguria (urine output ≤0.5 mL/kg/h) for ≥2 h despite optimized supportive measures such as adequate filling volumes, the use of high-dose catecholamines (epinephrine, dobutamine, or norepinephrine), and the insertion of an IABP. The hemodynamic criteria were systolic arterial pressure < 80 mm Hg, cardiac index less than 2.2 L/min/m^2^, and pulmonary capillary wedge pressure of at least 20 mm Hg. Patients over 18 years old at the time of VA ECMO treatment were included. The patients with a subclavian steal phenomenon or atherosclerotic disease of the carotid arteries (either side) according to preoperative Doppler-sonography were excluded. Figure 1 is a schematic diagram of the study protocol.

During the time of investigation, all of the patients continuously received inotropic drugs and/or vasopressors (epinephrine, norepinephrine, enoximone, dobutamine or dopamine) and nitroglycerine. To prevent risks, studies were performed only in the patients who could tolerate a brief period without IABP assistance after their circulatory condition was stabilized with VA ECMO support. All of the patients received midazolam for pain relief and sedation.

### Intra-aortic balloon pump

For the patients presenting with refractory cardiogenic shock who did not respond to fluid resuscitation or vasopressor infusion, an IABP was considered. The IABP was installed before VA ECMO support in those critically ill patients. In all of these patients, the IABP catheter used was a 7.5-F, 40-ml balloon Percor STAT-DL catheter (Datascope Corp, Fairfield, NJ) connected to a Datascope portable computerized console (Datascope), placed using a percutaneous insertion technique via the femoral artery. We positioned the distal tip of the balloon catheter in the descending thoracic aorta 2 cm distal to the origin of the left subclavian artery. The catheter position was confirmed by a chest x-ray. Balloon inflation was trigged by the R wave of the electrocardiogram. The balloon was inflated just before the diastolic notch of the arterial pressure waveform and deflated before ventricular systole. The electrocardiogram (ECG)-triggered IABP was set to a 1:1 assist mode and 100% balloon augmentation during VA ECMO support.

### ECMO circuit and management

Despite IABP support and high dosages of inotropic drugs (dobutamine, dopamine, and epinephrine), there was constant low cardiac output. VA ECMO is often considered if the patient remains unstable under IABP support. The decision to initiate ECMO was made by the attending cardiac surgeons, the ECMO specialist, and the ICU attending staff. The ECMO circuit consisted of a centrifugal pump console, a Rotaflow (Maquet, Jostra, Hirrlingen, Germany) in conjunction with a membrane oxgenator, a Quadrox D (Maquet, Jostra) with integrated heart exchanger, an oxygen/air blender, and an oximetry monitor. All of the components were heparin coated. For all of these patients, the ECMO cannulae (Biomedicus, Medtronic; Minneapolis, MN, USA) were inserted through the femoral artery and femoral vein via surgical cut down. We always used a small downflow cannula into the superficial femoral artery to ensure that the arterial flow to the leg was maintained.

To initiate VA ECMO, the flow rate in the circuit was increased gradually over a period of several minutes to 3.5 L/min to achieve a mixed venous oxygen saturation (SvO_2_) of 65% and a systolic blood pressure arterial pressure of 80-100 mm Hg. With full VA ECMO support, intravenous inotropes and vasopressors were reduced, and a protective lung ventilation strategy was applied to allow for optimal myocardial and pulmonary recoveries. The fractional inspired oxygen was adjusted to maintain a PO_2_ between 100 and 200 mmHg. Continuous intravenous heparin was administered to obtain an activated clotting time of 180-220 s during VA ECMO support.

The VA ECMO support was terminated once hemodynamic stability was restored with only minimal pharmacologic inotropic support. The blood gas was analyzed every 4 hours during the VA ECMO support. The routine blood counts and blood chemistries were analyzed every 12 hours. The patients’ hematocrit values were maintained at 30% to 50%, and platelets were transfused when the platelet count was less than 100 × 10^3^/mm^3^ during the VA ECMO support. The cardiac function was monitored by transthoracic echocardiography (a left ventricular ejection fraction ≥ 40%) during the weaning process. Step-by-step weaning was the main strategy. The VA ECMO flow rate was reduced to 1.5 L/min over a period of several hours. Peripheral cannulae were removed under direct vision at the bedside with formal repair of the vessels.

### Transcranial doppler ultrasound

The measurements were performed in the cardiac intensive care unit (ICU) of the Beijing Anzhen Hospital. The patients were attached to TCD (SONSRA, VIASYS, Healthcare Cor, USA) for CBF monitoring once every 12 hours. The Transcranial Doppler monitoring of the bilateral middle cerebral arteries was performed with two 2.5 MHz transducers fitted on a headband. The ultrasound probes were fixed around the patient’s head with a special elastic tape to minimize movement artifacts. The depth of insonation varied between 35 and 52 mm until representative spectral artery flow was identified. The Doppler ultrasound variables, including the depth, gain, sample volume and power of the ultrasound beam, were not changed during the study period. The CBFV was shown in cm/sec. The CBFV Transcranial Doppler signals were measured once every 12 hours under turned on IABP and turned off IABP support. After 5 minutes IABP stabilization, the CBFV was measured. The envelope curves of the Doppler frequency shift and figures were recorded digitally for a period of 30 seconds during each measurement and stored in a microcomputer for further off-line analysis. Using custom software, the data were analyzed off-line to calculate the mean CBF. The unilateral mean CBF was derived from integration over time. The bilateral mean CBFV was calculated using the built-in calculation program [CBFm = (left CBFm + right CBFm)/2]. The patients provided their control values. The CBF was affected by the cerebral perfusion pressure, the patient’s state of alertness, the hematocrit (Hct), the angle of insonation, and the vessel diameter [15]. The routine monitoring indicators, including the arterial blood pressure, continuous SvO_2_, the VA ECMO support flow rate, the arterial blood gases, and mean blood pressure, were kept unchanged during the entire study periods.

### Statistical analysis

The normally distributed data were reported as the mean ± standard deviation, and the abnormally distributed data were reported as median values with the interquartile range (IQR; 25th and 75th quartiles). The statistical analysis was performed on the absolute values, and each patient served as his or her own control. The paired *t* test was used to compare the mean of the parametric variables for VA ECMO with IABP and VA ECMO alone. The statistical analysis was performed using SPSS 19.0 software (SPSS Inc, Chicago, IL). A value of p < 0.05 was considered statistically significant for all of the variables.

## Results

### Clinical outcomes

Of the 12 patients, nine patients (75.0%) were male. The pre-operative ejection fraction was 42.6 ± 14.2% (29.5% to 58.8%). All of the patients received an IABP before VA ECMO implantation. The Sequential Organ Failure Assessment (SOFA) score was 12.2 ± 4.6. The inotropic score was 85.2 ± 62.9 μg/kg/min. The pre-ECMO blood gas values were: pH 7.26 ± 0.07; PaCO_2_ 38.4 ± 8.5 mmHg; PaO_2_ 75.1 ± 29.8 mmHg. The mean pre-ECMO blood lactate level was 10.9 ± 4.5 mmol/L. The peak creatinine kinase MB form (CK-MB) value was 335.8 ± 211.3 U/L. In these patients, the median time interval from the end of the operation to the initiation of VA ECMO was 16.5 ± 4.2 hours (range 8-25 hours).

The mean duration of VA ECMO support was 125.90 ± 42.18 hours (75 to 200 hours). All 12 adult patients could be successfully weaned from the VA ECMO. Eight patients were able to be discharged from the hospital. The overall survival rate was 66.7%. The mean IABP support time was 9.6 ± 3.7 days (range 5 to 15 days). The mean length of stay in the ICU was 13.2 ± 3.7 days (range 8-19 days). The causes of mortality in patients after weaning from VA ECMO support included multi-organ failure in 2 patients (16.7%) and sepsis in 2 patients (16.7%).

Bleeding and acute renal failure were the most common complications of the VA ECMO. Re-thoracotomy was required in 2 patients (16.7%) for bleeding or pericardial tamponade. Acute renal failure requiring continuous venovenous hemofiltration occurred in 2 (16.7%) patients. The means of the units of blood products transfused were 6.0 ± 1.4 units of packed red blood cells, 982.6 ± 129.2 ml of fresh frozen plasma, and 2.8 ± 1.6 units of platelets. IABP complications were not observed in our patients.

### Cerebral blood flow

No statistically significant hemodynamic changes occurred between the two groups (p > 0.05) (Table 1).

**Table 1:** Hemodynamic status details of the two groups

		**N group**	**P group**
heart rate	IABP	92.8 ± 9.6	100.0 ± 13.5
	turned off IABP	95.0 ± 13.5	97.8 ± 12.3
systolic pressure (mmHg)	IABP	90.8 ± 5.2	89.6 ± 5.4
	turned off IABP	89.6 ± 4,9	90.1 ± 4.8
diastolic pressure (mmHg)	IABP	79.1 ± 6.2	72.6 ± 5.7
	turned off IABP	77.6 ± 5.3	72.6 ± 6.8
mean artery pressure (mmHg)	IABP	82.6 ± 5.4	78.9 ± 5.4
	turned off IABP	81.6 ± 4.6	77.8 ± 5.7
oxygen saturation (%)	IABP	95.0 ± 2.6	94.7 ± 2.4
	turned off IABP	94.5 ± 2.4	94.4 ± 2.9
VA ECMO flow (L/min)		3.6 ± 0.2	3.4 ± 0.4
Hct (%)		29.4 ± 4.6	29.7 ± 4.3

No statistically significant hemodynamic changes occurred between the two groups (*p* > 0.05).

**Figure 2:**
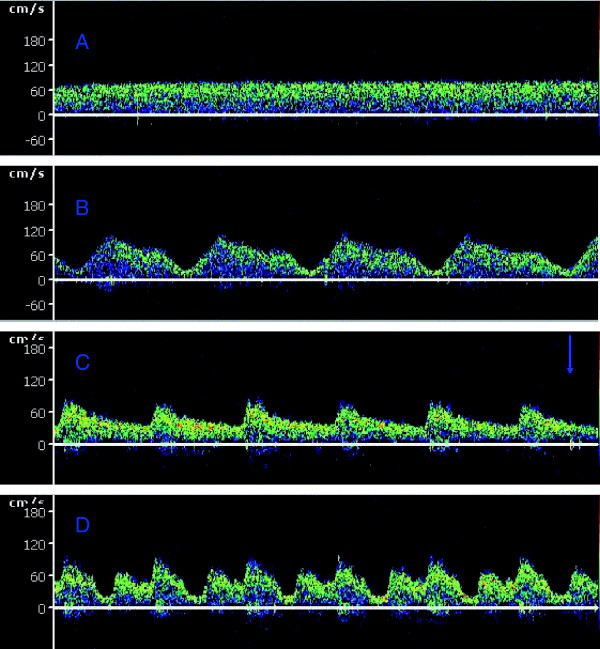
**Typical middle cerebral artery flow waveform obtained by transcranial Doppler ultrasonography during extracorporeal membrane oxygenation. A**, **B**, N group. **C**, **D**, P group. **(A)** The CBFV while IABP is turned off in severe cardiac failure. **(B)** The CBFV while ECMO was combined with IABP support in severe cardiac functional failure. **(C)** The CBEV while IABP is turned off in spontaneous cardiac function. **(D)** The CBFV while ECMO was combined with IABP support in spontaneous cardiac function.

**Figure 3:**
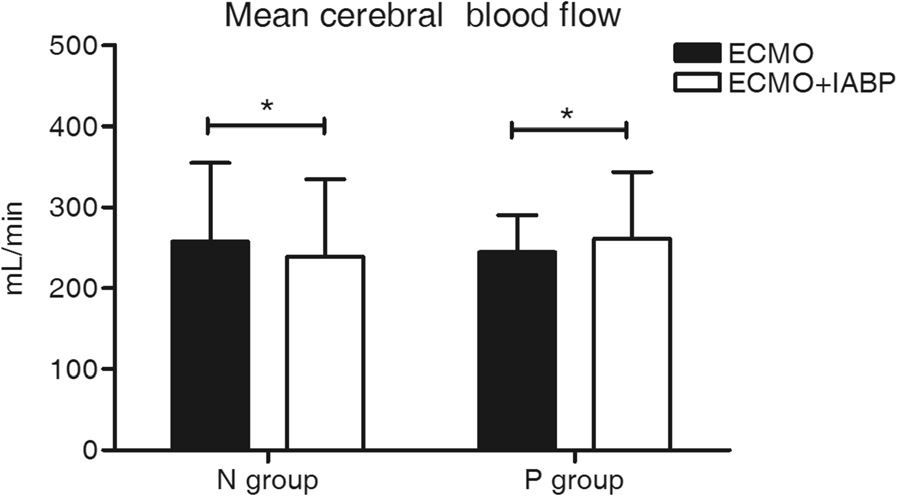
**Mean CBF in the N group and the P group under ECMO with IABP.** An IABP significantly decreased the mean CBF during non-pulsatile pressure ECMO support. An IABP significantly increased the mean CBF during pulsatile pressure ECMO support. The results are reported as the mean ± standard. *, *p* < 0.01 between the IABP support data and the data without IABP support.

Figure 2 shows a typical middle cerebral artery blood flow waveform obtained by transcranial Doppler ultrasonography with turned on IABP and turned off IABP during VA ECMO support. One hundred and thirty pairs of data were obtained during VA ECMO support. No statistically significant differences for the mean CBFs were observed from the patients between on VA ECMO alone and on VA ECMO with IABP support (251.47 ± 79.28 ml/min vs. 251.30 ± 79.47 ml/min, *P* = 0.96). Considering the cardiac functional state, the data were divided into two groups (pulsate pressure over 10 mmHg, P group, n = 85; pulsate pressure lower than 10 mmHg, N group, n = 45) by the pulsate pressure without IABP support. The addition of an IABP to VA ECMO significantly decreased the mean CBF in the N group (257.68 ± 97.21 ml/min vs. 239.47 ± 95.60 ml/min, *P* = 0.00). The addition of an IABP to VA ECMO support led to a significant increase in the mean CBF values in the P group (261.68 ± 82.45 ml/min vs. 244.43 ± 45.85 ml/min, *P* = 0.00) (Figure 3 and Table 2).

**Table 2:** Outcome of the CBF in the patients

	**Total**	**N group**	**P group**
VA ECMO with IABP (ml/min)	251.47 ± 79.28	239.47 ± 95.60	261.68 ± 82.45
VA ECMO alone (turned off IABP) (ml/min)	251.30 ± 79.47	257.68 ± 97.21	244.43 ± 45.85
*p* value	0.96	0.00	0.00

## Discussion

VA ECMO is effective for the treatment of acute cardiac failure, however, the neurological morbidity has become a significant concern for many patients [16]. At one academic medical center, combined ischemic and hemorrhagic stroke affected 7% of the adult patients supported with ECMO and significantly increased the odds ratio of death [17]. Brain death occurred in 7% to 21% of the cases of ECMO-treated adults in some ECMO centers [6]. In 2009, the national Extracorporeal Life Support Organization (ELSO) registry reported that 21% of 295 adults treated with ECPR experienced brain death. Additionally, approximately one-half of the survival patients showed evidence of cerebral injury [8]. CBF reduction (inadequate perfusion) and non-pulsate blood flow during VA ECMO might play a role in the pathogenesis of this complication [8,9].

VA ECMO and IABP are well-established treatments in patients suffering postcardiotomy cardiogenic shock [12]. Most clinicians ignored the critical question of brain perfusion during the combination of these two treatments. There are many reports regarding the effect of IABP on cerebral circulation [8,18-21]. The outcomes are unclear. In a study of animal models with cardiogenic shock, when the cardiac output was reduced by one-third and the cerebral perfusion was reduced by 80%, the IABP support caused a further 10% reduction in the CBF [18]. In human studies, Gee and colleagues [19] noted an overall reduction of 11.6% in the ocular blood flow measured by ocular pneumoplethysmography in 56 patients on IABP assistance. Similarly, a reverse CBF occurring at the end of the diastole was observed by Schachtrupp and colleagues [9]. In a model of VA ECMO-treated cardiac arrest, the authors tested an often encountered clinical combination of VA ECMO with IABP. They found that when used with the femoro-femoral VA ECMO approach, IABP did not significantly change the CBFV [20]. A clinical study reported that because of an early systolic reversal blood flow, no net increase occurred in the total flow in the common carotid artery with IABP support [21]. These results indicated that rapid deflation of the IABP decreased the pre-ejection CBF, and cerebral perfusion might be inadequate or unchanged. In our study, although this type of reverse phenomenon was not observed in any of the patients, the outcome might be dependent on the antegrade blood flow. And this phenomenon was supported by the results from Schachtrupp and colleagues [9] reported that IABP significantly increased the antegrade mean flow in the middle cerebral artery in patients after cardiac surgery.

During the peripheral VA ECMO support, the blood ejected from the heart theoretically would perfuse the heart, brain, and upper extremities. Taylor and colleagues reported that the CBF was determined by the cardiac function by analyzing the effects of the systolic cardiac performance on the intracranial hemodynamics in nine infants undergoing ECMO [22]. Rosenberg and Kinsella, using the radioactive microsphere method, showed that the CBF velocity was decreased in newborn lambs after the initiation of VA ECMO support at low flow rates. Higher flow rates could stabilize cerebral hemodynamics [23]. These findings suggested that CBF is dependent on cardiac output or the intensity of the ECMO support.

IABP was employed in most of the VA ECMO setups with the aim of reducing the after load to improve the coronary perfusion and maintain a pulsatile blood flow. Previous studies have suggested an improved clinical outcome of VA ECMO support combined with IABP in patients with cardiogenic shock [12]. There are concerns that an intermittent aortic occlusion by the IABP balloon might diminish the available cerebral blood [13]. No studies have reported the cerebral flow during the concomitant support of VA ECMO and IABP in adults by TCD, as was done in this study. We found that the mean CBF was significantly lower in VA ECMO combined with IABP support than in VA ECMO alone during myocardial stunning (a pulsate pressure lower than 10 mmHg) when there was slight antegrade flow ejected by the heart. We found that the CBF values were significantly higher in VA ECMO combined with IABP than with VA ECMO alone during the spontaneous cardiac function period (Figure 2). The effect of IABP on CBF during VA ECMO support appears to vary with the spontaneous cardiac function state.

No significant differences in our study were found in the response of the mean CBFs during the combination support and VA ECMO. The authors commented as to the specific condition between the statement of the spontaneous cardiac and that of the VA ECMO and IABP support. The effect of IABP on the CBF might be different for severe cardiac failure and spontaneous cardiac function during VA ECMO support. When the data were analyzed between the different cardiac functional states, the data of the mean CBF were divided into two groups (P group and N group) by the cardiac function state (determined with pulsate pressure after turning off IABP during the VA ECMO support). These results demonstrated that in a severe cardiac dysfunction situation (a pulsate pressure lower than 10 mmHg), IABP might have an unfavorable effect on CBF during the peripheral VA ECMO support. The implementation of IABP during the early stage of the ECMO support resulted in a significant decrease of the CBF. Clinically, some patients had essentially no ventricular cardiac output with ‘ventricular stun’ during VA ECMO support. The pulse pressure of the pericallosal artery is frequently lower than 10 mmHg [24]. The TCD ultrasonogram of the cerebral blood velocity tracing showed a flat waveform, as shown in Figure 2A. Under such conditions, VA ECMO provided retrograde blood flow from descending to ascending across the IABP. The diastolic inflation of the intra-aortic balloon might intermittently compromise the retrograde non-pulsatile ECMO flow to the brain during the concomitant use of ECMO and IABP. This finding might explain the reason that IABP in late diastole caused a drop in the CBFV during severe cardiac failure. The recovery of the heart results from the appearance of the antegrade aortic flow during VA ECMO support. The addition of IABP significantly increased the mean CBF under such conditions. Additionally, the use of an IABP to maintain pulsatility during VA ECMO support is uniformly agreed upon in some studies [11,13]. The pulsatility effect of IABP probably led to improved CBF perfusion and contributes to cerebral autoregulation recovery.

There are several limitations in our study. First, given the emergent setting, we lacked a neurological evaluation and CBF measurement performed prior to a trial of VA ECMO. Second, the state of the global and regional cerebral arteries was not examined in this study. Third, we did not study the long-term effects of the IABP and VA ECMO combination in our patients. The relationships between the CBF change and the neurological outcome of VA ECMO in adults are unknown.

## Conclusion

IABP significantly changed the CBF during peripheral VA ECMO, depending on the antegrade blood flow by spontaneous cardiac function. Our study showed that IABP significantly decreased the mean of the CBF during myocardial stunning, but increasing the mean of the CBF during the recovery of cardiac function.

## Abbreviations

IABP: Intra-aortic balloon pump; VA ECMO: Venoarterial extracorporeal membrane oxygenation; ECPR: Extracorporeal cardiopulmonary resuscitation; CBF: Cerebral blood flow; TCD: Transcranial doppler ultrasound; CBFV: Cerebral blood flow velocity; ECG: Electrocardiogram; Svo2: Mixed venous oxygen saturation; ICU: Intensive care unit; CPB: Cardiopulmonary bypass; ELSO: Extracorporeal Life Support Organization.

## Competing interests

The authors declare that they have no competing interests.

## Authors’ contributions

FY designed the experiments, analyzed and interpreted the data, and wrote the manuscript. ZSJ performed the experiments and analyzed and interpreted the data. LJX, XH, and CJJ provided the specimens and drafted the manuscript. ZW and YL performed the experiments, analyzed and interpreted data, and drafted the manuscript. XTH conceived the study, designed and interpreted the data and drafted the manuscript. FY, ZSJ, HW, and MJ critically discussed the paper. All of the authors gave their final approval for the submission of the manuscript.
